# Reports on Hospitals of the United Kingdom

**Published:** 1914-02-07

**Authors:** Henry Burdett


### REPORTS ON
### Hospitals of the United Kingdom.

By SIR HENRY BURDETT, K.C.B., K.C.V.O.

SERIES III.

### BEDFORD COUNTY HOSPITAL.

This hospital was established in 1802. The present hospital consists entirely of new buildings, which were erected in 1890 from plans by Mr. Percy Adams, and are, we were informed, the first hospital buildings erected by him. The plan is that adopted by several of the larger hospitals and Poor-Law infirmaries erected on the pavilion plan. The ward pavilions are on one side of the corridor, and on the other is situated the out-patient department, a spacious and amply planned structure with a central waiting hall, with an operation block, which when erected was imperfect and is now being reconstructed so as to provide an anæsthetising room and a thoroughly and properly fitted sterilising section. The theatre is much too large, judged by the later types, but it is well equipped and thoroughly well lighted artificially, though we imagine that on dull days the ground-glass windows may make the natural light faulty.

In front of the ward and other blocks is placed the administration building, which contains any number of small rooms, and strange to say the kitchen department is none too large. Why the kitchen unit should have been selected for economising space in a plan where place allotment is sometimes so excessive we are at a loss to understand. The wards are well proportioned and contain sixteen beds, placed in two-storey pavilions, each containing forty beds, being arranged in two large wards and two small ones. The space allotted to the w.c.s and sinks is too restricted, and there is no room for mackintosh sinks, so that they have to be washed in the baths—a very objectionable practice. The committee's liberality, of which there is abundant evidence, will no doubt lead them to sanction the provision of mackintosh sinks, which will have to be placed in the bathrooms, where there is ample room. The bed-pans are kept in racks in the lavatory section, and as these racks are placed immediately above the heating coil it would be an improvement to close the racks in, on the Norwich plan, in such a way as to retain sufficient heat to keep the bed-pans at a temperature ready for immediate use. The interspace between the corridor and the wards is unusually great, and contains a considerable number of departments, including one for patients' clothes—the best provision of the kind we have seen anywhere—a larder, a separate coal cellar on the ground floor, with the sick ward for nurses and a number of other small sections, each self-contained and each entered by a separate door.

There is, however, no sisters' room, and the nurses use a very ample day room attached to each ward, which is seldom used by the patients, owing to the fact that as soon as patients are convalescent they are discharged. The children's ward block is well fitted, and the condition of the children demonstrates the efficiency of the sister in charge of it, who has, we imagine, no easy task owing to the admission of a number of very young children as well as those of a more manageable age. Having regard to the fact that each sister is responsible for four wards, containing altogether forty beds, we are of opinion that they should be paid not less than £40 a year each; the sisters' work and responsibility are by no means light, and the excellent order of the wards and other departments warrants our calling attention to this point.

The chapel, with its stained-glass windows and carved altar, is a notable feature of the hospital, but the altar rail, which seems to have been an afterthought, constructed as it is of wood, is very heavy and out of character with the altar. It is also in the way of the lectern. There is an attractive board room, which contains an ancient Turkey carpet, showing some æsthetic taste on the part of the board, and possibly a wish not to be extravagant, so far as the furnishing of this room is concerned. The medical library, where the honorary staff are able to hold meetings, is an interesting and roomy chamber containing a number of books, some of which are of historical interest, as are some of the portraits to be found on its walls. On the ground floor adjacent to the board room is a special room for the chairman, the officers' dining-room, and the secretary's office. Upstairs are the quarters of the resident medical staff; they have each a roomy and attractive sitting-room, as well as unusually good bedrooms. Opposite their quarters are those of the matron, whose office adjoins her sitting-room, and her quarters are attractive too. The bathrooms in this section of the hospital are excellent, as are those in the wards.

There is an isolation block; a laundry, which has recently been fitted up with new and modern machinery, and a mortuary that is defective for the reason that it contains no chapel or view room. Nor is it constructed and decorated so as to display that reverence for the dead which in our view is so important for moral and educational reasons. The elevation of this hospital is attractive. We congratulate the committee on the interest they take in everything connected with this institution, which reflects credit upon the town and county.

The secretary was unfortunately away, our visit being paid on Saturday afternoon, October 25, 1913. We are exceedingly indebted to the Matron, Miss A. Charteris, who took care that we should see every portion of the building and every detail of its administration. We congratulate her on her department and on the condition of the stores and other sections which come more directly under her charge. She is evidently interested in her work, and possesses the great merits of energy and a desire to bring every portion of the hospital up to the highest point of efficiency.

**The Northampton General Hospital will be reported on next week.**

